# The role of patient navigation programs in early cancer care in Mexico: a multi-case qualitative study

**DOI:** 10.1093/oncolo/oyaf314

**Published:** 2025-09-27

**Authors:** Elysse Bautista-González, Yanin Chavarri-Guerra, Anne Peasey, Hynek Pikhart, Cecilia Vindrola-Padros

**Affiliations:** Research Department of Epidemiology and Public Health, Institute of Epidemiology and Health Care, University College London, London WC1E 7HB, United Kingdom; Departamento de Hemato-Oncología, Instituto Nacional de Ciencias Médicas y Nutrición Salvador Zubirán (INCMNSZ), Ciudad de México 14080, México; Research Department of Epidemiology and Public Health, Institute of Epidemiology and Health Care, University College London, London WC1E 7HB, United Kingdom; Research Department of Epidemiology and Public Health, Institute of Epidemiology and Health Care, University College London, London WC1E 7HB, United Kingdom; Rapid Research Evaluation and Appraisal Lab (RREAL), Department of Targeted Intervention, University College London, London WC1E 6BT, United Kingdom

**Keywords:** patient navigation programs, cancer, Mexico

## Abstract

**Background:**

In Mexico, academic publications on patient navigation are notably scarce. Thus, limited evidence in Mexico suggests that patient navigation programs (PNP) may play a promising role in early cancer care. The study’s aim is to identify and describe PNP in Mexico, particularly their role in early diagnosis and opportune treatment.

**Methods:**

Through an exploratory qualitative cross-sectional case study design. Five different programs were identified using snowball sampling. Thematic guides were developed. Data were collected through funnel-shaped semi-structured interviews with patient navigation providers. After familiarizing with the identified themes, codes were generated inductively.

**Results:**

PNP in Mexico navigate 1 or multiple types of cancer patients, using heterogeneous sources of funding, navigate 1 or multiple levels of healthcare and from within or outside of the healthcare system; they aim to improve access to healthcare, address barriers, and reduce wait times. However, PNP often engage in activities that are not aligned with their objectives. In assessing their impact, disparities are not measured, and no data are collected at time intervals.

**Conclusion:**

Using theoretical frameworks and logic models can support the implementation of new PNP, guide early diagnosis and treatment outcome measurement, and assess impact—ultimately helping ensure financial sustainability.

Implications for Practice:

1. Patient navigation programs (PNP) in Mexico are heterogeneous and adapt to the changing healthcare access landscape across the cancer continuum.
2. Upon the design of new PNP in Mexico, stakeholders must clearly identify where in the pathway to treatment they act upon and at which levels of care, before measuring outcomes.
3. While PNP help patients overcome barriers, they rarely track whether the support actually reduces inequalities or shortens the time to diagnosis or treatment. PNP in Mexico must operationalize outcome measurements using available frameworks.
4. PNP in Mexico must design interventions targeting minorities and ensure evaluation of disparities in outcomes in their research agenda.
5. It is imperative to identify new and sustainable ways to maintain PNP activities in the long term.

## Background

Patient navigation programs (PNP) were developed in the United States to overcome barriers to cancer care.^1^ Studies in low- and middle-income countries (LMICS) have shown that PNP can improve access to healthcare, reduce health disparities, and increase the proportion of patients receiving appropriate cancer care^2^^,^^3^ and reducing delays in care.^2^^,^^4^^,^^5^

In Mexico, cancer represents a significant challenge for the healthcare system, being one of the main causes of morbidity and mortality.^6^ Issues such as late diagnoses and treatment,^7–9^ inequalities in access to healthcare^6^^,^^7^^,^^10^ and resources for diagnosis and treatment have been previously highlighted.^11^^,^^12^ However, until now, only 2 PNP publications have been found related to breast cancer.^13^^,^^14^ One successfully reduced referral times for specialized cancer care among low-income patients,^13^ treatment initiation time of 33 days from the first contact with the program.^14^ Nevertheless, it is crucial to describe other PNP in the country navigating other cancer patients, as well as patients potentially being navigated outside of the health system, in primary care or secondary care, detailing their characteristics, populations served, activities performed, and the impact metrics used to determine their role in early diagnosis and treatment.

## Methods

A qualitative cross-sectional case study design was used to investigate unstudied PNP in Mexico and comparing their characteristics.^15^^,^^16^ Research took place during the development of the principal investigator’s (E.B.G.) doctoral fieldwork conducted from January to March 2019. Five different PNP were identified in Mexico using the snowball sampling technique in order to identify individuals or organizations that might not label themselves as a PNP but were still relevant to the study according to other organizations. As part of the exploratory work, this method is commonly used to reach “hidden” groups that are difficult to access through traditional sampling methods. The first program was identified based on the E.B.G. prior knowledge, nonetheless she had no prior relationship with the interviewees. The inclusion criteria for the PNP required that they have at least the 4 fundamental elements of patient navigation support: case identification, barrier detection, development of a personalized plan, and systematic follow-up. The members of the PNP decided who would be interviewed: either the navigator or the program director. One member from each PNP was recruited, informed about the research objective, and, if interested, signed the consent form to participate in the study. The interviews were conducted at the facilities of the PNP and were carried out by E.B.G.

Semi-structured interviews were used to assess details such as program origins, populations served, disease focus, objectives, available resources, activities performed, evaluation mechanisms, and the monitoring of health disparities throughout the disease process. Thematic guides were developed to structure the conversation between the researcher and the PNP representative. These guides were based on a literature review and reviewed by the second author (C.V.P.). The interviews were recorded in audio format and had an average duration of 1 hour. The transcripts were imported into NVivo and after an initial stage of familiarization with the collected data, codes were labelled inductively by E.B.G. and revised by C.V.P. The study did not require approval from the university’s ethics committee.^17^ All those invited to the study agreed to participate in the interviews. In total, members of 5 PNP were interviewed. The study was based on the COREQ criteria for qualitative studies^18^ and the case study methodology.^16^^,^^19^^,^^20^

## Results

All 5 PNP interviewed were implemented from 2010 onwards. The PNP studied supported a diverse range of patients, with the number of newly diagnosed patients per year being between 500 and 1100, including those without health insurance, with private insurance and with public insurance. Meanwhile, some were part of the public health sector, others were independent non-governmental organizations (NGOs). PNP implement their programs in clinical (hospital-based) or community settings. Program navigators had diverse professional backgrounds, including nurses, health care professionals (doctors), psychologists, social workers, and cancer survivors. All PNP studied assist cancer patients, although some focus on specific types of cancer such as lung or breast, while others navigate patients with multiple cancer types and at different stages of the cancer continuum. PNP studies had different objectives, and navigation involved a diverse set of activities. Table 1 shows the summary of the 5 case studies and Table 2 shows the activity codes found in each program.

**Table 1.** Summary of the patient navigation program case studies.

Characteristics	Case study A	Case study B	Case study C	Case study D	Case study E
**Origin**	2013	2010	2013	2014	2015
**Patients navigated**	500/year	330/year	1100/year	100/year	400/year
**Region**	Chiapas	National	Mainly central Mexico: Mexico City, Mexico State, Hidalgo, Puebla	National	National
**Target population**	Uninsured patients in Indigenous regions	Uninsured cancer patients	Uninsured or insured cancer patients	Uninsured women under 40 with breast cancer	Uninsured with lung cancer
**Type of cancer**	All types	All types (mainly breast cancer)	Lung, prostate, testicular, breast, ovarian, cervical, and hematological cancers	Breast cancer only	Lung cancer only
**Setting**	Community-based (rural)	Hospital-based (urban)	Community and hospital-based (urban)	Hospital-based (urban)	Hospital-based (urban)
**Type of organization**	Non-governmental organization (NGO)	Public health institution	Non-governmental organization (NGO)	Non-governmental organization (NGO)	Public health institution
**Main objective**	Facilitate access to quality healthcare in uninsured Indigenous communities	Improve the cancer care experience at the hospital level	Address economic barriers to improve access to cancer diagnosis and treatment	Facilitate access to services not covered by the public institution, such as fertility preservation and breast reconstruction	Reduce time to treatment initiation for hospitalized lung cancer patients
**Type of navigator**	Social worker, physicians, driver	Peer navigator, psychologist, nurse	Social worker	Psychologists and physicians	Nurse
**Main activities**	Identifying barriers, transport, emotional support, appointment management, coordination, mediation, funding	Telephone communication, emotional support, appointment management, mediation, support groups, post-treatment job reintegration	Appointment management, mediation, emotional support, cancer treatment funding, connecting with external resources	Emotional support, mediation, administrative support, connecting with external resources	Appointment management, clinic attendance, mediation during appointments, linking to external resources
**Method of communication**	Telephone, WhatsApp	Telephone, direct messaging, hospital hotline, website, WhatsApp	Social media, telephone, WhatsApp	Social media, telephone, WhatsApp	Telephone, WhatsApp
**Impact evaluation**	Barrier quantification, number of patients assisted	Patient satisfaction, barrier quantification, survival follow-up (planned)	Barrier quantification, number of patients assisted	Patient satisfaction, quality of life evaluation, psychological assessment	Number of patients assisted

**Table 2.** Summary of patient navigation program case studies coded activities.

		A	B	C	D	E
**1. Navigation by health-care system levels**						
** Health-care level navigated**	From primary care level to 2nd or 3^rd^	YES				
	From 2nd level to 3^rd^			YES		
	Within 3rd level		YES		YES	YES
**2. Basic navigation activities**						
** Patient identification**	Activity related to the active search of eligible patients	YES	YES	YES	YES	YES
** Barriers and resource identification**	Identification of barriers in access to healthcare.	YES	YES	YES	YES	YES
	Activity related to the identification of resources already found in the patient’s context.	YES	YES	YES	YES	YES
** Continuous evaluation of barriers**	Based on previous barriers, a continuous evaluation of barriers is conducted.			YES		
	Active identification of new barriers			YES		
**3. Specific interventions/activities**						
** Introduction to environment**	Teach patients how to navigate the hospital and/or health sector	YES	YES	YES		YES
** Administrative documentation**	Provision of support to fill internal documentation	YES	YES	YES	YES	
	Provision of support to fill external documentation	YES	YES	YES		
** Appointment management**	Schedule appointment with the medical team	YES	YES	YES	YES	YES
	Appointment reminders for all the appointments, including the first	YES				
** Mediation between doctor and patient**	Communication between the medical team and the patient	YES	YES		YES	YES
** Donation of resources**	Donation of cancer treatment		YES	YES		
	Donation of food			YES		
	Donation of transportation to travel to hospital/clinic	YES		YES		
	Donation of shelters/hotel stay during the patients	YES				
	Donation of diagnostic (lab-tests) and treatment (not cancer related)	YES		YES	YES	YES
** Linkage with external resources**	Connection with state/public transportation services and/or shelter services	YES	YES	YES		YES
	Connection to other resources from non-profits or other organizations		YES	YES	YES	YES
	Access to discounts for hotel, medicines, transportation and food	YES	YES	YES		
	Legal advice			YES		
	Job re-integration support		YES			
	Wellness activities		YES			
** Provision of information**	Provision of information on cancer, diagnosis, treatment, survival and other cancer related topics	YES	YES	YES		YES
	Information on which external resources are available	YES	YES	YES	YES	
	Communication with the patient throughout the navigation experience	YES	YES	YES	YES	
** Psychological support**	Create and administer support group		YES	YES	YES	
	Generate a direct communication line with the navigator for emotional support		YES	YES	YES	
	Sexual health therapy				YES	
	Psychological therapy				YES	

### Case study A

A social worker, in collaboration with the medical doctor and a taxi driver are trained to navigate the patient. The social worker and doctor identify the barriers and match them with interventions at the community level. The taxi driver transports the patient to the closest hospital, interprets for the patient if necessary and mediates with the doctor to reach appointments sooner. Thereafter, the social worker communicates with the patient through telephone or WhatsApp. The intervention activities include introducing the indigenous patient to the health system environment, aid in administrative tasks (ie, filling documentation in Spanish), appointment management, mediation between the doctor and uninsured ethnic minority patients. Due to the nature of the organization, this navigation program not only linked patients with other collaborators (ie, other NGO, donors), but also donated resources geared to tackle economic barriers because of transportation hurdles and shelter access difficulties. In some cases, this PNP also donated diagnostic procedures (ie, cancer confirmation in private clinic). Additionally, a key objective within this PNP is the provision of information with regards to diagnosis, treatment, and close relationship with the patient throughout the cancer continuum. This is mainly done by the navigator in close relationship with the patient’s physician. Although this PNP did not systematically include psychological services as an activity, mental health services were always available through another program.

### Case study B

The navigator communicates with the uninsured patients through telephone, direct messaging, and a specific hospital line to aid the patient reach a greater understanding of their disease. Their activities included: introducing all cancer patients to the hospital environment, maintaining a personalized and friendly environment, aid in administrative tasks (ie, filling documentation), appointment management and mediation between the doctor and patient. They tackled economic barriers mainly through collaborations with external resources (ie, free regional transportation, discounts in hotels, food, medicines, and diagnostic procedures). The provision of information and emotional support for all cancer patients is crucial. Therefore, this PNP has a direct line for patients, an educational website, and a psychological support group for each type of cancer. In addition, the patient can directly speak to the navigator for emotional support. After being treated, patients are supported through wellness and work reintegration programs.

### Case study C

They communicate with the patient through social media, telephone line, and WhatsApp. After identifying barriers, this PNP continuously evaluates the barriers being tackled and re-evaluates barriers through-out the cancer continuum. This NGO introduces the patient both to the health-system and hospital environments. They aid in administrative tasks such as filling documentation or appointment management. To tackle economic barriers, this PNP not only donates food and diagnostic tests, but actively funds cancer treatment. Additionally, they also link the patient to external resources (ie, state transportation, other NGOs, legal services).

### Case study D

Navigators are psychologists and communicate with the patient through social media, telephone line and WhatsApp. Based in Mexico City, they help the patient with some administrative, logistical, mediation and linkage with external resources tasks, however, these are not their core objectives. This PNP navigates the patient in the hospital environment and mainly provides emotional support and psychological therapy to breast cancer patients under the age of 40. This privately funded organization also donates private diagnostic services and treatment for some patients.

### Case study E

The navigator introduces the patient to the clinic, managing their appointments, and mediating when these are not suitable for the patient. This PNP donates lung cancer treatment for the uninsured population through the acquisition of grants. In addition, they provide the patient with information on cancer and link the patient with external resources to tackle personal barriers to care. Although this PNP did not systematically include psychological services as an activity, mental health services were always available through another clinic.

## Discussion

These case studies captured programs that have been developed in the last 10 years in various regions of Mexico with the purpose of guiding people to access care. The navigated population presents different insurance coverage schemes; there is heterogeneity across cases in the approach to cancer, objectives, resources used, financing, and evaluation methods. The PNP studies aim to intervene along the cancer continuum, in different time intervals and all seek to help those who are most at risk of delaying or not accessing care or those who are at risk of catastrophic expenses. However, over time, these programs discuss they have evolved to address changing challenges, adopting various emerging activities.

### Navigation across the cancer continuum in tiered health care systems

**Figure 1.**
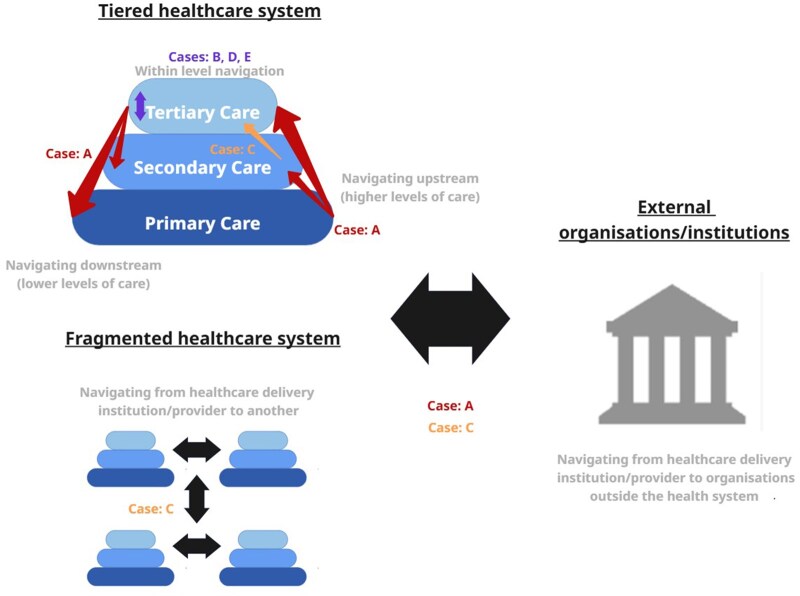
Graphic representation of PNP activities within and outside the health sector. PNP, patient navigation programs.

In the literature, PNP usually thrive in diverse care settings, spanning hospitals, community health centers, mobile clinics, and even platforms. This adaptability aims for accessibility and seamless navigation for patients.^21–23^ In the context of Mexico’s healthcare system, characterized by 3 levels of care,^24–28^ this study reveals the challenge of navigating patients (upstream, downstream and within a single level of care) throughoutthe fragmented healthcare landscape. Figure 1 shows a graphic representation of the types of navigation taking place in the case studies: either in the health system itself, or outside the health system, within a single healthcare institution or different ones, sometimes at more than one level of care delivery. Although the PNP studied typically adapt their services based on available resources and patient characteristics, this analysis adds a new dimension: the starting point of a patient’s navigation journey within the healthcare system, and whether that navigation is initiated internally by the system itself. This perspective challenges conventional ways of measuring navigation—often based on simply counting activities^29^^,^^30^—and instead argues that the true intensity of navigation is better reflected by the range of healthcare levels involved and the complexity of processes addressed across the cancer care continuum.^31^

### Patient navigator background and training

In the literature, PNP vary in the professionals they employ. Some programs seek out cancer survivors as navigators,^32^ and sometimes navigators are nurses, health professionals, social workers, or community representatives with little or no previous experience in the medical field and no clinical training. Some studies also report a mix of patient navigator professional backgrounds. Other programs go beyond professional background and may also seek to employ navigators with race and language concordance to their patients’ characteristics in order to increase the effectiveness of the program.^33^^,^^34^

According to these 5 case studies, patient navigators in Mexico include cancer survivors, individuals with sociodemographic characteristics similar to the patients, and community members.^2^ However, 1 case stands out: a driver served as a navigator in an indigenous region. This inclusive and flexible approach suggests that individuals with communication skills and a willingness to help can play a key role in improving access to healthcare.

Formal training for navigators has proven to be essential to achieve desired outcomes and different training material has been developed over time.^35^ Content such as basic health promotion, privacy, end of life, advanced directives, and visit guides are some examples of the content developed to train navigators.^36^ Despite this, the availability of formal patient navigation training courses is limited in both Mexico and Latin America, and most of these courses are offered only occasionally. For instance, the National Cancer Institute used to offer a virtual course for healthcare professionals or civil society volunteers, but it is currently inactive.^37^ On the other hand, the National Institute of Medical Sciences and Nutrition Salvador Zubirán offered a Patient Navigation educational program through a virtual extension for community healthcare outcomes (ECHOS) model for healthcare professionals.^37^ This course demonstrated a significant increase in knowledge about patient navigation, and participants reported feeling significantly more prepared to manage the barriers faced by patients and institutions.

Standardizing training helps reduce health barriers and disparities among different patient groups.^38^ The American Cancer Society recommends implementing patient navigation educational programs to standardize professional knowledge, directly contributing to reducing health barriers and disparities. This has a positive impact on cancer patients by expanding access to high-quality navigation programs.^38^

### Patient navigation beyond the health system

Literature suggests navigators may be linked to resources outside the health system (ie, other healthcare providers, social services, and community programs).^39^^,^^40^ They connect patients to community-based programs to help overcome their personal barriers, including proactively connecting patients to external resources, following patients after referral, and providing information and encouragement.^41^ Navigators in these case studies also play a central role in establishing connections beyond the health system. Their proactive activities involve linking patients with healthcare providers, social services, and community programs, employing a holistic approach to overcome personal barriers and provide ongoing support after referral.

The PNP studies have demonstrated their ability to adapt to the changing health landscape. With survival increasing thanks to innovative therapies, they are working to ensure access to treatments. However, financial barriers pose significant challenges, adjusting fundraising strategies to cover expensive treatments and provide ongoing support. To illustrate, while breast cancer treatment is completely covered by public insurance, the same is not true for lung cancer.^10^ This financial disparity highlights the need for greater resources to guide patients with certain types of cancer. As barriers change and health coverage evolves, interventions targeting navigated populations also transform, prioritizing specific aspects and adapting to patients’ changing needs. As some therapeutic regimens have slowly been fully covered by the health system, patient navigation has shifted toward psychological, logistical, or other barriers. This ability to adapt highlights the critical importance of PNP in improving the patient experience in the healthcare system.

### Frameworks and missed opportunities

In the literature, some PNP focus on navigating patients with a single cancer type^42–45^ or multiple types of cancer.^40^^,^^46^^,^^47^ This study presents organizations in which patients with different types of cancer are navigated simultaneously, through heterogeneous funding sources and diverse approaches. In the future, it is important to conduct research studies to evaluate the impact between programs that navigate multiple types of cancer versus those that focus on a single type of cancer.

The case studies take on activities that are not aligned with their objectives, evaluation indicators that do not cover all activities, or use basic indicators designed primarily for administrative purposes. Some programs evaluate patient satisfaction, quality of life along the cancer continuum, and psychological evaluations. However, a significant gap exists as none of them have collected data on time intervals to diagnosis or treatment, leading to a lack of evidence demonstrating a reduction in delays in cancer patient care due to the implementation of PNP. In the international literature, PNP have also failed to evaluate long-term impact, particularly in terms of time-to-event intervals.^21^^,^^48–51^.

In comparison with Alerta Rosa in Monterrey^14^ and the breast cancer PNP in Mexico City, these 5 case studies, there appears to be no clear integration between the patient navigation research agenda and early diagnosis and timely treatment. Scarcity of human and economic resources could explain the limited evaluation efforts and small presence of PNP in the academic realm. This raises questions about limitations imposed by organizational structures and formalization of PNP in the health system.

### Health equity evaluation and impact

The 5 PNP studied have not implemented measures to reduce disparities in outcomes within their populations. Patient navigation is distinguished from other services by its focus on reducing health inequalities.^52^ To fulfil their mission, they must address individual barriers and design interventions that eliminate disparities among different groups. They can achieve this by using available guidelines to develop equity-focused healthcare interventions.^53–56^ The literature provides good examples of PNP integrating interventions to reduce health disparities.^53^

### Sustainability and financial mechanisms

This study reveals that only 2 PNP in Mexico receive public funds. Most depend on private financing, obtaining support from grants, donations, and collaborations with private entities. This diversity of funding introduces different organizational dynamics, impacting objectives, scope, and sustainability. The interaction between organizational dynamics and financing models is complex, providing support to patients. Despite limited resources, these are good examples of non-public financing. Ultimately, securing funding would be more feasible if PNP demonstrate positive impacts on cancer care.

As an exploratory study, these results do not represent the full navigation spectrum that might exist in Mexico and current reality needs to be explored. A systematic mapping of all PNP in Mexico has not yet been carried out and therefore is encouraged for future research and evaluations.

## Conclusion

These results contribute to the understanding of the PNP in Mexico. PNP are recommended to use theoretical frameworks and tools to evaluate their objectives, goals and activities. They could also employ a logic model to operationalize their results and evaluate their intervention. It is crucial to train PNP in the generation of evidence to facilitate decision-making about its inclusion as a strategy for reducing time intervals in cancer management.

## Data Availability

The data underlying this article are available in the article.

## References

[1] Freeman HP , Rodriguez RL. History and principles of patient navigation. Cancer. 2011;117:3539-3542. 21780088 10.1002/cncr.26262 PMC4557777

[2] Dalton M , Holzman E, Erwin E, et al Patient navigation services for cancer care in low-and middle-income countries: a scoping review. PLoS One. 2019;14:e0223537. 31622363 10.1371/journal.pone.0223537 PMC6797131

[3] Louart S , Bonnet E, Ridde V. Is patient navigation a solution to the problem of “leaving no one behind”? a scoping review of evidence from low-income countries. Health Policy Plan. 2021;36:101-116. 33212491 10.1093/heapol/czaa093 PMC7938515

[4] Hoffman HJ , LaVerda NL, Young HA, et al Patient navigation significantly reduces delays in breast cancer diagnosis in the District of Columbia. Cancer Epidemiol Biomarkers Prev. 2012;21:1655-1663. 23045540 10.1158/1055-9965.EPI-12-0479 PMC6615038

[5] Chan RJ , Milch VE, Crawford-Williams F, et al Patient navigation across the cancer care continuum: an overview of systematic reviews and emerging literature. CA Cancer J Clin. 2023;73:565-589. 37358040 10.3322/caac.21788

[6] Goss PE , Lee BL, Badovinac-Crnjevic T, et al Planning cancer control in Latin America and the Caribbean. Lancet Oncol. 2013;14:391-436. 23628188 10.1016/S1470-2045(13)70048-2

[7] Bautista-Gonzalez E , Hasselkus-Sánchez G. Barriers and facilitators of early lung cancer care in Mexico: a qualitative exploration from patients, relatives, and health professionals. GAMO. 2025;24:1-14.

[8] Unger-Saldaña K , Miranda A, Zarco-Espinosa G, Mainero-Ratchelous F, Bargalló-Rocha E, Miguel Lázaro-León J. Health system delay and its effect on clinical stage of breast cancer: multicenter study. Cancer. 2015;121:2198-2206. 25809536 10.1002/cncr.29331 PMC6681165

[9] Unger-Saldaña K , Ventosa-Santaulària D, Miranda A, Verduzco-Bustos G. Barriers and explanatory mechanisms of delays in the patient and diagnosis intervals of care for breast cancer in Mexico. Oncologist. 2018;23:440-453. 29284758 10.1634/theoncologist.2017-0431 PMC5896704

[10] Gerson R , Zatarain-Barrón ZL, Blanco C, Arrieta O. Access to lung cancer therapy in the Mexican population: opportunities for reducing inequity within the health system. Salud Publica Mex. 2019;61:352-358. 31276352 10.21149/10118

[11] Ferlay J , Ervik M, Lam F, *Global Cancer Observatory: Cancer Today (Version 1.1) [Internet]*. International Agency for Research on Cancer; 2024. https://gco.iarc.who.int/today (Accessed: 15/04/2024)

[12] Bautista-Gonzalez E, Quintero Leyra A, Munoz Rocha TV, et al. Assessing disparities in cancer resources distribution in Mexico. BMC Health Serv Res. 2025;25:564. 10.1186/s12913-025-12497-z PMC12007217 40247345

[13] Soto-Perez-de-Celis E , Chavarri-Guerra Y, Ramos-Lopez WA, et al Patient navigation to improve early access to supportive care for patients with advanced cancer in resource-limited settings: a randomized controlled trial. Oncologist. 2021;26:157-164. 33210345 10.1002/onco.13599 PMC7873328

[14] Mireles-Aguilar T , Tamez-Salazar J, Muñoz-Lozano JF, et al Alerta Rosa: novel alert and navigation breast cancer program in Nuevo Leon, Mexico, for reducing health system interval delays. Oncologist. 2018;23:1461-1466. 30126860 10.1634/theoncologist.2018-0226 PMC6292552

[15] Baxter P , Jack S. Qualitative case study methodology: study design and implementation for novice researchers. *TQR*. 2015;13:554–559.

[16] Stake RE. The Art of Case Study Research. 1st ed. Sage Publications; 1995.

[17] University College London. *Do I Need Ethical Approval? [Internet].* University College London Ethics; 2024. Accessed February 01, 2019. https://www.ucl.ac.uk/research-ethics/do-i-need-ethical-approval#:∼:text=You%20do%20not%20need%20to, be%20classed%20as%20’research’

[18] Tong A , Sainsbury P, Craig J. Consolidated criteria for reporting qualitative research (COREQ): a 32-item checklist for interviews and focus groups. Int J Qual Health Care. 2007;19:349-357. 17872937 10.1093/intqhc/mzm042

[19] Yin RK. Case Study Research and Applications: Design and Methods. 6th ed. Sage Publications, Inc.; 2018.

[20] Budgell B. Guidelines to the writing of case studies. J Can Chiropr Assoc. 2008;52:199-204. 19066690 PMC2597880

[21] Freund KM , Battaglia TA, Calhoun E, et al National cancer institute patient navigation research program: methods, protocol, and measures. Cancer. 2008;113:3391-3399. 18951521 10.1002/cncr.23960 PMC2698219

[22] Freund KM. Patient navigation: the promise to reduce health disparities. J Gen Intern Med. 2011;26:110-112. 21161422 10.1007/s11606-010-1593-5 PMC3019331

[23] Hou S-I , Roberson K. A systematic review on US-based community health navigator (CHN) interventions for cancer screening promotion—comparing community- versus clinic-based navigator models. J Cancer Educ. 2015;30:173-186. 25219543 10.1007/s13187-014-0723-x

[24] Juan M , Moguel Ancheita A, Valdés Olmedo C, Universalidad de los servicios de salud en México. *Salud Publica Mex*. 2013;55:1-64. 24570037

[25] Gómez-Dantés O , Sesma S, Becerril VM, Knaul FM, Arreola H, Frenk J. Sistema de salud de México. Salud Publica Mex. 2011;53:S220-S232. 21877087

[26] Knaul FM , Arreola-Ornelas H, Touchton M, et al Setbacks in the quest for universal health coverage in Mexico: polarised politics, policy upheaval, and pandemic disruption. Lancet. 2023;402:731-746. 37562419 10.1016/S0140-6736(23)00777-8

[27] Gómez-Dantés O , Frenk J. Chronicle of a century of public health in Mexico: from public health to social protection in health. Salud Publica Mex. 2019;61:202-211. 30958963 10.21149/10122

[28] Frenk J , Gómez-Dantés O, Knaul FM. A dark day for universal health coverage. Lancet. 2019;393:301-303. 30661745 10.1016/S0140-6736(19)30118-7

[29] Carroll JK , Winters PC, Purnell JQ, Devine K, Fiscella K. Do navigators’ estimates of navigation intensity predict navigation time for cancer care? J Cancer Educ. 2011;26:761-766. 21556957 10.1007/s13187-011-0234-y PMC4401038

[30] Glassgow AE , Molina Y, Kim S, Campbell RT, Darnell J, Calhoun EA. A comparison of different intensities of patient navigation after abnormal mammography. Health Promot Pract. 2019;20:914-921. 29907079 10.1177/1524839918782168 PMC6274628

[31] Walter F , Webster A, Scott S, Emery J. The Andersen model of total patient delay: a systematic review of its application in cancer diagnosis. J Health Serv Res Policy. 2012;17:110-118. 22008712 10.1258/jhsrp.2011.010113 PMC3336942

[32] Psooy BJ , Schreuer D, Borgaonkar J, Caines JS. Patient navigation: improving timeliness in the diagnosis of breast abnormalities—ProQuest. Can Assoc Radiol J. 2004;55:145-150. 15237774

[33] Tan CHH , Wilson S, McConigley R. Experiences of cancer patients in a patient navigation program: a qualitative systematic review. JBI Database Syst Rev Implement Rep. 2015;13:136-168. 10.11124/jbisrir-2015-1588 26447039

[34] Charlot M , Santana MC, Chen CA, et al Impact of patient and navigator race and language concordance on care after cancer screening abnormalities. Cancer. 2015;121:1477-1483. 25565151 10.1002/cncr.29221 PMC4409461

[35] Ustjanauskas AE , Bredice M, Nuhaily S, Kath L, Wells KJ. Training in patient navigation: a review of the research literature. Health Promot Pract. 2016;17:373-381. 26656600 10.1177/1524839915616362 PMC4899310

[36] Fink RM , Kline DM, Siler S, Fischer SM. Apoyo con cariño: a qualitative analysis of a palliative care-focused lay patient navigation intervention for Hispanics with advanced cancer. J Hosp Palliat Nurs. 2020;22:335-346. 32568935 10.1097/NJH.0000000000000666

[37] Chavarri Guerra Y , Ramos-Lopez WA, Olaya-Vargas A, et al Patient navigation educational curriculum for cancer care in Mexico. JCO. 2023;41:e18592.

[38] American Cancer Society. *Leadership in Oncology Navigation [Internet]*. American Cancer Society; 2024. Accessed May 01, 2024. https://www.cancer.org/health-care-professionals/resources-for-professionals/patient-navigator-training.html#:∼:text=Training%20and%20credentialing%20will%20help, disparities%20among%20various%20patient%20groups

[39] Ko N , Bak S, Nelson K, et al A patient-centered approach to a cancer care delivery innovation for low income patients: medical-legal partnership with patient navigation. *J Clin Oncol.* 2015;33:15.

[40] Battaglia TA , Gunn CM, Bak SM, et al Patient navigation to address sociolegal barriers for patients with cancer: a comparative-effectiveness study. Cancer. 2022;128 Suppl 13:2623-2635. 35699610 10.1002/cncr.33965 PMC10152516

[41] Loskutova NY , Tsai AG, Fisher EB, et al Patient navigators connecting patients to community resources to improve diabetes outcomes. J Am Board Fam Med. 2016;29:78-89. 26769880 10.3122/jabfm.2016.01.150048

[42] Gilbert J , Veazie S, Joines K, et al Patient Navigation Models for Lung Cancer. Agency for Healthcare Research and Quality (US); 2018. 30586255

[43] Langballe R , Dalton SO, Jakobsen E, et al NAVIGATE: improving survival in vulnerable patients with lung cancer through nurse navigation, symptom monitoring and exercise—study protocol for a multicentre randomised controlled trial. BMJ Open. 2022;12:e060242. 10.1136/bmjopen-2021-060242 PMC9628541 36316074

[44] Myers RE , Sifri R, Daskalakis C, et al Increasing colon cancer screening in primary care among African Americans. J Natl Cancer Inst. 2014;106. 10.1093/jnci/dju344 PMC4817126 25481829

[45] Christie J , Itzkowitz S, Lihau-Nkanza I, Castillo A, Redd W, Jandorf L. A randomized controlled trial using patient navigation to increase colonoscopy screening among low-income minorities. J Natl Med Assoc. 2008;100:278-284. 18390020 10.1016/s0027-9684(15)31240-2

[46] Cykert S , Eng E, Manning MA, et al A multi-faceted intervention aimed at Black-White disparities in the treatment of early stage cancers: the ACCURE pragmatic quality improvement trial. J Natl Med Assoc. 2020;112:468-477. 30928088 10.1016/j.jnma.2019.03.001

[47] Fleisher L , Miller SM, Crookes D, et al Implementation of a theory-based, non-clinical patient navigator program to address barriers in an Urban Cancer Center setting. J Oncol Navig Surviv. 2012;3:14-23. 25383260 PMC4222195

[48] Gorin SS , Haggstrom D, Han PKJ, Fairfield KM, Krebs P, Clauser SB. Cancer care coordination: a systematic review and meta-analysis of over 30 years of empirical studies. Ann Behav Med. 2017;51:532-546. 28685390 10.1007/s12160-017-9876-2

[49] Guadagnolo BA , Dohan D, Raich P. Metrics for evaluating patient navigation during cancer diagnosis and treatment: crafting a policy-relevant research agenda for patient navigation in cancer care. Cancer. 2011;117:3565-3574. 21780091 10.1002/cncr.26269 PMC4818009

[50] Crane-Okada R. Evaluation and outcome measures in patient navigation. Semin Oncol Nurs. 2013;29:128-140. 23651682 10.1016/j.soncn.2013.02.008

[51] Lee J-H , Fulp W, Wells KJ, Meade CD, Calcano E, Roetzheim R. Effect of patient navigation on time to diagnostic resolution among patients with colorectal cancer-related abnormalities. J Cancer Educ. 2014;29:144-150. 24113902 10.1007/s13187-013-0561-2 PMC3945676

[52] Hendren S , Griggs JJ, Epstein RM, et al Study protocol: a randomized controlled trial of patient navigation-activation to reduce cancer health disparities. BMC Cancer. 2010;10:551. 20939928 10.1186/1471-2407-10-551 PMC2964637

[53] Ward PR , Meyer SB, Verity F, Gill TK, Luong TCN. Complex problems require complex solutions: the utility of social quality theory for addressing the social determinants of health. BMC Public Health. 2011;11:630. 21819576 10.1186/1471-2458-11-630 PMC3167771

[54] Masseria C , Hernández-Quevedo C, Allin S. Health inequality: what does it mean and how can we measure it? Expert Rev Pharmacoecon Outcomes Res. 2010;10:177-186. 20384564 10.1586/erp.10.14

[55] Asada Y , Hurley J, Norheim OF, Johri M. A three-stage approach to measuring health inequalities and inequities. Int J Equity Health. 2014;13:98. 25366343 10.1186/s12939-014-0098-y PMC4222403

[56] Alleyne GAO , Castillo-Salgado C, Schneider MC, Loyola E, Vidaurre M. Overview of social inequalities in health in the region of the Americas, using various methodological approaches. Rev Panam Salud Publica. 2002;12:388-397. 12690726 10.1590/s1020-49892002001200005

